# A R2R3-MYB Transcription Factor of *GmMYB62* Regulates Seed-Coat Color and Seed Size in Arabidopsis

**DOI:** 10.3390/ijms26083457

**Published:** 2025-04-08

**Authors:** Bi-Yao Zhao, Jian-Bo Yuan, Jin-Bao Gu, Cong Li, Yan Lin, Yu-Hang Zhang, Bai-Hong Zhang, Yin-Hua Wang, Xing Ye, Yang Li, Zhen-Yu Wang, Tian-Xiu Zhong

**Affiliations:** 1 Department of Grassland Science, College of Forestry and Landscape Architecture, South China Agricultural University, Guangzhou 510642, China; 13623678585@163.com (B.-Y.Z.); wyinhua24@163.com (Y.-H.W.); yx33058605@163.com (X.Y.); 2 Institute of Nanfan & Seed Industry, Guangdong Academy of Science, Guangzhou 510316, China; yuanjingan113@163.com (J.-B.Y.); gjbxx2578@163.com (J.-B.G.); licong202103@163.com (C.L.); linyan_sher@163.com (Y.L.); yuhangzhangedu@126.com (Y.-H.Z.); zhangbyhome@126.com (B.-H.Z.); yangli111208@126.com (Y.L.); 3 Zhanjiang Research Center, Institute of Nanfan & Seed Industry, Guangdong Academy of Science, Zhanjiang 440000, China

**Keywords:** soybean, seed-coat color, seed size, MYB, anthocyanin

## Abstract

The seed-coat color and seed size have an impact on both the evolutionary fitness and the grain yield of crops. Soybean is a major oil crop, and the seed-coat color and seed size exhibit natural diversity among the different soybean varieties. Here, we found an R2R3-MYB transcription factor of *GmMYB62*, which shows a significant increase in expression as the seed-coat color changes from yellow to black in different soybean varieties. The *GmMYB62* was specifically highly expressed in reproductive organs, especially in floral organs in soybeans. The *GmMYB62* encodes a nuclear protein that contains two MYB domains. In the phylogenetic analysis, the GmMYB62 was relatively conserved after the divergence of the monocots and dicots, and it also grouped with transcriptional repressors of MYBs in anthocyanin synthesis. The *GmMYB62* was overexpressed in Arabidopsis and the seeds displayed a pale-brown coat in *GmMYB62* overexpression lines, in contrast to the dark-brown seed coat observed in wild-type of Col-0. The anthocyanin content in the *GmMYB62* overexpression lines was dramatically reduced when compared to Col-0. Additionally, the seeds in overexpression lines showed shorter lengths, larger widths, and lower thousand-seed weights than those in Col-0. Furthermore, the genes related to anthocyanin synthesis and seed size regulation were investigated, and expression of eight genes that involved in anthocyanin synthesis pathway, like *chalcone synthase* (*CHS*), *chalcone isomerase* (*CHI*), *flavanone 3-hydroxylase* (*F3H*), and *anthocyanidin synthase* (*ANS*) were severely inhibited in the *GmMYB62* overexpression lines when compared to Col-0. In addition, the *ARGOS-LIKE* (*ARL*), *B-Type Cyclin 1* (*CYCB1*), and *enhancer of DA1-1* (*EOD3*), which govern cell expansion and proliferation, were highly expressed in *GmMYB62* overexpression lines when compared to Col-0. Overall, this study sheds new light on the control of seed-coat color and seed size by *GmMYB62* and provides potentially valuable targets for improving crop seed quality.

## 1. Introduction

Soybean [*Glycine max* (L.) Merr.], is one of the most crucial oil crops worldwide [1,2]. There is a huge demand for soybean production due to its content of approximately 20% oil and 40% protein in the seeds [3,4,5]. The soybean seed coat presents a range of colors, including yellow, brown, purple red, black, and so on, and it is an important agronomic trait and an evolutionary characteristic [6]. The black/brown seeds usually accumulate a higher content of anthocyanins and flavonoids in the seed coat compared to yellow seeds. The flavonoids and anthocyanins have attracted widespread attention in the market due to their antioxidant properties and flavors. The seed-coat color is an evolutionary feature in the soybean subgenus, and it changed from black to various colors during the domestication of wild soybeans into cultivated soybeans [6]. The seed weight is one of the most essential yield-related traits in soybean [7,8], and it is affected by many environmental and genetic factors during the soybean growth and development stages [9]. Therefore, identifying novel genes governing seed weight is important for soybean genetic improvement and high-yield breeding [10,11,12].

The seed-coat color is an obvious quantitative agronomic trait in soybean. The genes responsible for seed-coat color were also involved in other functions, including the seed-coat cleavage, the seed-coat expansion, and seed dormancy in soybeans [13]. There are multiple genes controlling seed-coat color that have been studied in soybeans. The *chalcone synthase* (*CHS*) encoded the key enzymes in the flavonoid and anthocyanin synthesis to regulate seed-coat color in soybean, and nine homologous were found in the soybean genome [14,15,16]. The *ARGONAUTE* (*AGO5*) and *DICER-LIKE* (*DCL2a/b*) control the soybean seed-coat color by producing small interfering RNAs from long inverted repeats in *CHS* genes [17]. As the key factor in the anthocyanin synthesis pathway, the loss-function of *flavonoid 3′-hydroxylase* (*F3′H*) resulted in the change from brown to grey in soybean seed-coat color [18]. A *myeloblastosis* (*MYB*) transcription factor of *Glyma.09G235100* governs the seed-coat color by upregulating expression of anthocyanidin synthase (*ANS*) genes to promote the anthocyanins synthesis in soybean [19,20]. The *chalcone isomerase 1A/2* (*CHI1A/2*), working for flavonoid biosynthesis, was also implicated in seed-coat color regulation in soybean [19].

There are also multiple genes identified for the regulation of seed size in soybeans. The *GmSWEET10a/b* (*SWEET family of sugar transporters*) was involved in soybean seed oil content and size/weight by controlling sugar partitioning from seed coat to embryo [21]. The soybean mutant with loss function of *GmKIX8-1* (*KIX domain-containing protein*) produced seeds with bigger size [9]. The expression of *GmBS1/2* (*BIG SEEDS*) was down-regulated by artificial microRNA to increase the seed size/weight in soybeans [22]. In addition, the natural allelic variations in *SW16.1* (*Seed Weight*), *GmST05* (*Seed Thickness 05*)*, POWR1* (*Protein*, *Oil*, *Weight*, *Regulator 1*), *GmGA3ox1* (*GA 3b-hydroxylase*), and *GmCYP82C4* (*Cytochrome P450s*) also affect seed size in soybean [10,11,12,23,24].

The anthocyanins are responsible for the diverse colors of leaves, flowers, fruits, and seeds in plants [25,26]. The R2R3 MYBs are a group of plant-specific transcription factors that regulate the expression of genes involved in anthocyanin and flavonoid biosynthesis, which are indispensable for the development and stress resistance in plants [26]. The *LvMYB5* and *LvMYB1* improved the anthocyanin biosynthesis level in lily flowers by increasing the expression levels of *CHS*, *dihydro favonol-4-reductase* (*DFR*), and *ANS* genes [27]. In Arabidopsis, the synthesis of proanthocyanidins in the seed-coat was controlled by *AtMYB123/TT2* [28]. Overexpression of pear (*Pyrus bretschneideri*) *PbMYB1L* induced significant anthocyanin accumulation in leaves and high expression of anthocyanin structural genes in Arabidopsis [29]. The *FaMYB5* was involved in the MYB-bHLH-WD40 (MBW) activation complex to positively regulate the biosynthesis of anthocyanin in strawberries [30]. However, a balloon flower (*Platycodon grandiflorus*) PlgMYBR1, acts as a negative regulator for anthocyanin biosynthesis by inhibiting the function of *AtPAP1* (*Production of anthocyanin pigment 1*) in Arabidopsis [31]. The high accumulation of anthocyanin occurred in *Atmyb3* mutant plants, and the negative effect was caused by AtMYB3 through its function as a transcriptional repressor in the anthocyanin biosynthesis pathway [32]. The banana *MaMYB4* and grape VvMYBC2-L1 also play roles as repressors in the anthocyanin or proanthocyanidin biosynthesis pathway [33,34]. Therefore, anthocyanin synthesis is co-regulated by positive and negative transcription factors of MYBs, and identification of novel MYBs in anthocyanin biosynthesis is essential for flower color and seed-coat color research in plants. Moreover, the MYBs involved in anthocyanin formation or suppression could be identified and classified by phylogenetic analysis in plants [35].

In soybean, the GmMYBA5, GmMYBA2, and GmMYBA1 induced anthocyanin accumulation by upregulating the expression of anthocyanin pathway-related genes [36]. The *GmMYB114* (*Glyma.17G143600*) is in response to blue light exposure, and overexpression of *GmMYB114* in soybean hairy roots substantially promoted anthocyanin synthesis [37]. Moreover, knockout *GmMYB77* (*Glyma.04g036700*) significantly increased isoflavone content, while its overexpression resulted in a serious decrease in isoflavone content in soybean [38]. In addition, the *GmMYB78* (*Glyma.10G010300*) enhances soybean sensitivity to *Phytophthora sojae* by repressing the jasmonic acid signaling pathway and the expression of pathogenesis-related genes [39]. Overexpression of *GmMYB183* in Arabidopsis and soybean hairy roots improved plant aluminum tolerance with higher citrate secretion [40]. Here, we identified an R2R3-MYB transcription factor of *GmMYB62*; its expression highly increases with the seed-coat color from yellow to black in different soybean lines. For the soybean seeds with the same coat color (brown or black), the expression of *GmMYB62* in smaller seeds was also higher than in bigger seeds. The *GmMYB62* encodes a nuclear protein with two MYB domains and is grouped with transcriptional repressors of MYBs in phylogenetic analysis. In order to analyze the specific function of *GmMYB62* in plants, overexpression of *GmMYB62* in Arabidopsis was performed, and the seed-coat color, seed size, and the genes involved in anthocyanin biosynthesis were also investigated in transgenic Arabidopsis.

## 2. Results

### 2.1. The Expression of GmMYB62 Continues to Increase in Soybean Seed-Coat from Yellow to Black

**Figure 1:**
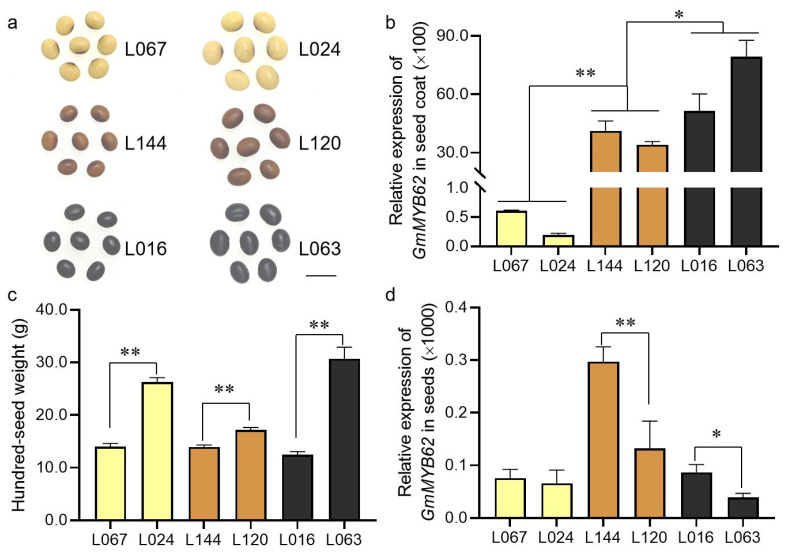
Phenotypes of soybean seeds and quantitative real-time polymerase chain reaction (qRT-PCR) analysis of the *GmMYB62* gene in seeds and seed coat. (**a**) Phenotypes of seed size and seed-coat color in six soybean varieties. (**b**) Relative expression of *GmMYB62* in soybean seed coat. (**c**) Hundred-seed weight in six soybean varieties. (**d**) Relative expression of *GmMYB62* in soybean seeds. Scale bar: 1.0 cm. Data represents the mean ± standard deviation of three replicates. Significant differences (* *p* < 0.05; ** *p* < 0.01) were determined by Student *t*-tests.

The R2R3-MYB transcription factors have been reported to play important roles in the regulation of seed-coat color and seed size in plants. Here, we selected six soybean accessions with yellow, brown, and black seed coats and different seed sizes (Figure 1a). When the expression of *GmMYB62* was detected in soybean seed-coat, the result showed that the *GmMYB62* expression highly increased over 50-fold with the seed-coat color from yellow to black (Figure 1b). The significant difference in hundred-seed weight, seed length, and seed width was also detected in soybean accessions with the same seed-coat color (Figure 1 and Figure S1). The expression of *GmMYB62* was also detected in soybean seeds, and it was highly expressed in small brown seeds and small black seeds when compared to big brown seeds and big black seeds, respectively (Figure 1d). However, no significant difference in *GmMYB62* expression was found between yellow seeds with different sizes (Figure 1d). These results suggested that the *GmMYB62* plays a crucial role in regulating seed-coat color and seed size in soybeans.

### 2.2. Expression Pattern and Phylogenetic Analysis of GmMYB62

**Figure 2:**
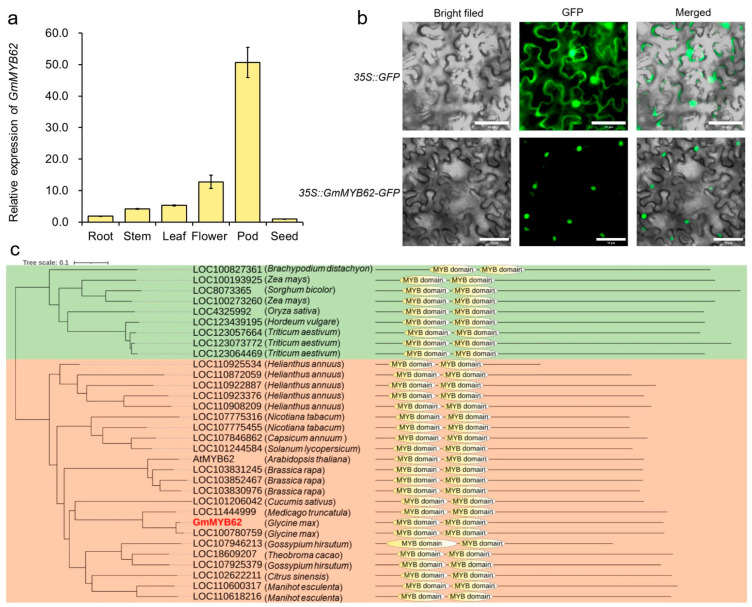
Expression pattern and phylogenetic analysis of GmMYB62 (**a**) Expression pattern of GmMYB62 in soybean tissues. *GmEF-1α* was used as a housekeeping gene. Data represent the mean ± standard deviation of three replicates. (**b**) The subcellular localization of GmMYB62 protein. *35S::GmMYB62-GFP* represents the tobacco leaves transformed by *pBin-GmMYB62-GFP* vector, and *35S::GFP* represents the empty vector (*pBinGFP4*). (**c**) Phylogenetic analysis of GmMYB62 and other 31 R2R3-MYB proteins from 19 species. The phylogenetic tree was constructed using the Neighbor-Joining method with 1000 bootstrap replicates using the MEGA version 11. All MYB protein sequences can be found in the NCBI (https://www.ncbi.nlm.nih.gov/, accessed on 23 March 2024) using the gene symbol or ID in the tree.

The expression level of *GmMYB62* in different soybean tissues was detected to reveal the tissue specificity and possible function of the target gene. The result found that *GmMYB62* presented lower expression in root, stem, and leaf, but higher expression in flower and pod in soybean (Figure 2a). To investigate the subcellular localization of GmMYB62, the CDS of *GmMYB62* without stop codon was fused to the GFP gene and expressed into the leaf epidermal cells of tobacco plants. As expected, the fluorescence from *35S::GFP* was ubiquitously distributed in the nucleus and cytoplasm, whereas the fluorescence from *35S::GmMYB62-GFP* was detected exclusively in the nucleus of cells (Figure 2b).

Conservative domain analysis revealed that there were two conserved domains (repeat R/MYB) at the N-terminus. The amino acid sequence at positions 19–69 was the R2 conserved domain, and the amino acid sequence at positions 72–120 was the R3 conserved domain, indicating that GmMYB62 belonged to the R2R3-MYB subfamily (Figure 2c). To elucidate the evolutionary relationship of *MYB* genes, a phylogenetic tree was constructed using the amino acid sequences of MYBs from soybean, Arabidopsis *thaliana*, rice (*Oryza sativa*), wheat (*Triticum aestivum*), and other 15 plant species. The soybean GmMYB62 was mainly grouped with MYBs in *Arabidopsis thaliana*, *Medicago truncatula*, *Gossypium hirsutum*, *Nicotiana tabacum*, etc. (Figure 2c). However, the MYBs from *Oryza sativa*, *Zea mays*, *Triticum aestivum*, and *Sorghum bicolor* were also clustered together into another tree branch (Figure 2c). The phylogenetic analysis indicated that the R2R3-MYBs are more conserved after the divergence of the monocot and dicot. Moreover, the GmMYB62 was also phylogenetically related to VvMYBC2-L1 and BaMYB4 as the repressors in another phylogenetic tree, which are negative regulators of anthocyanin biosynthesis and accumulation (Figure S2). This result suggests that the R2R3-MYB transcription factor of GmMYB62 is likely a repressor of anthocyanin biosynthesis, which could act with other MYB activators to balance anthocyanin accumulation in soybean seed coat.

### 2.3. Generation and Confirmation of GmMYB62-Overexpressing Transgenic Arabidopsis Plants

**Figure 3:**
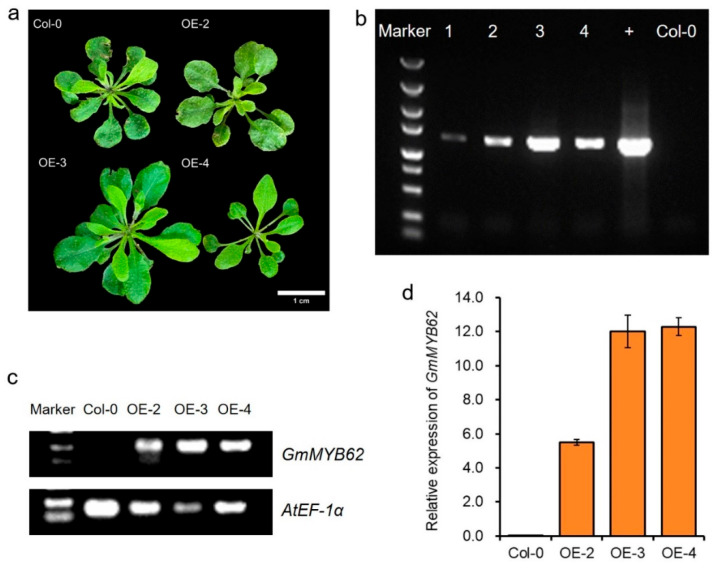
Generation and selection of *GmMYB62*-overexpressing transgenic *Arabidopsis thaliana*. (**a**) The Arabidopsis plants of Col-0 and *GmMYB62* overexpression lines. Scale bar: 1.0 cm. (**b**) Identification of T_1_ generation of *GmMYB62* transgenic *Arabidopsis thaliana* by polymerase chain reaction (PCR). 1–4 represent the transgenic lines. The + represents positive control and plasmid of *GmMYB62* vector was used as the template in PCR. (**c**) Identification of T_3_ generation of *GmMYB62* transgenic *Arabidopsis thaliana* by semi-quantitation reverse transcription PCR (RT-PCR). The endogenous gene of *AtEF-1α* was used as a control. (**d**) The relative expression levels of *GmMYB62* in the Col-0 and the *GmMYB62* transgenic Arabidopsis plants. OE-2/3/4 represents *GmMYB62* overexpression lines 2, 3, and 4, respectively. The *AtEF-1α* was used as a housekeeping gene in quantitative reverse transcription PCR (qRT-PCR). Data represents the mean ± standard deviation of three replicates.

Genetic transformation of *GmMYB62* into Arabidopsis plants was performed by the floral dip method, and the resistant genetically transformed seedlings were screened using a kanamycin screening medium (Figure 3a and Figure S3). Leaf DNA from transformed seedlings of *Arabidopsis thaliana* was extracted for PCR, and the *GmMYB62* transgenic lines were identified by 1.00% agarose gel electrophoresis with Col-0 DNA as the negative control and *GmMYB62* plasmids as the positive control (Figure 3b). In the RT-PCR result, the endogenous gene *AtEF-1α* was detected in all Col-0 and the transgenic lines, but the bands of *GmMYB62* were only detected in the *GmMYB62* overexpression lines (OE-2, OE-3, and OE-4) (Figure 3c). To further verify the expression level of *GmMYB62* in transgenic lines, the qRT-PCR was performed in Arabidopsis plants, and the transgenic lines showed significantly higher expression of *GmMYB62* than in Col-0 (Figure 3d).

### 2.4. Overexpression of GmMYB62 Regulates Seed-Coat Color and Seed Size in Arabidopsis

**Figure 4:**
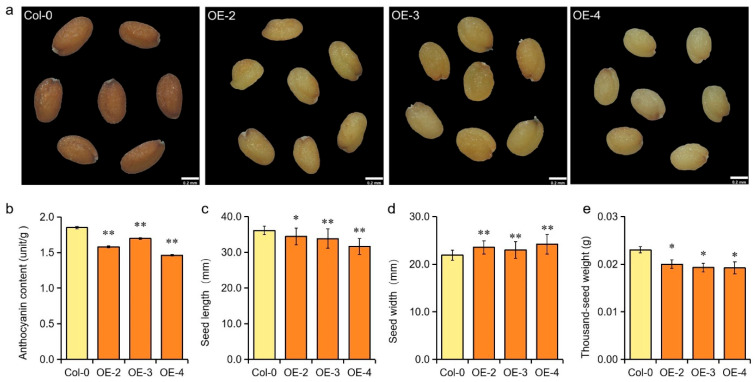
Phenotypes of seed-coat color and seed size in Col-0 and *GmMYB62* overexpression lines. (**a**) The seed-coat colors of the Col-0 and *GmMYB62* overexpression lines. Pictures were taken under a ZEISS microscope connected to a digital camera. Scale bars: 0.2 mm. (**b**) The anthocyanin content in the Col-0 and *GmMYB62* overexpression lines. (**c**) The seed length in the Col-0 and *GmMYB62* overexpression lines. (**d**) The seed width in the Col-0 and *GmMYB62* overexpression lines. (**e**) The thousand-seed weight in the Col-0 and *GmMYB62* overexpression lines. OE-2/3/4 represents *GmMYB62* overexpression lines 2, 3, and 4, respectively. Data represents the mean ± standard deviation of three replicates. Significant differences (* *p* < 0.05; ** *p* < 0.01) were determined by Student *t*-tests.

Accumulation of abundant anthocyanin in the seed coat will cause a brown color in Arabidopsis seeds. To test whether *GmMYB62* had a positive or negative effect on anthocyanin accumulation and seed-coat pigmentation, the seed-coat color was observed in Col-0 and *GmMYB62* overexpression lines. The Col-0 plants produced seeds with a dark-brown color, whereas the *GmMYB62* overexpression lines had seeds with a pale-brown color (Figure 4a). To further validate the downregulation effect of the GmMYB62 on anthocyanin accumulation, we measured the anthocyanin content in Col-0 and *GmMYB62* transgenic Arabidopsis plants. The results showed that the anthocyanin content in *GmMYB62* overexpression lines was significantly lower than that in Col-0 (Figure 4b).

Interestingly, the *GmMYB62* overexpression lines showed shorter seed length with an average 7.83% reduction and larger seed width with an average 7.13% increase compared to Col-0, respectively (Figure 4c,d). When the seed weight was measured, the *GmMYB62* overexpression lines presented a lower thousand-seed weight with an average 15.22% reduction compared to Col-0 (Figure 4e). These results indicated that *GmMYB62* works as a repressor in the anthocyanin accumulation to govern the seed-coat color, and it was also involved in the seed size regulation in Arabidopsis.

### 2.5. GmMYB62 Affects the Expression of Genes Related to Anthocyanin Biosynthesis and Seed Size Regulation in Arabidopsis

**Figure 5:**
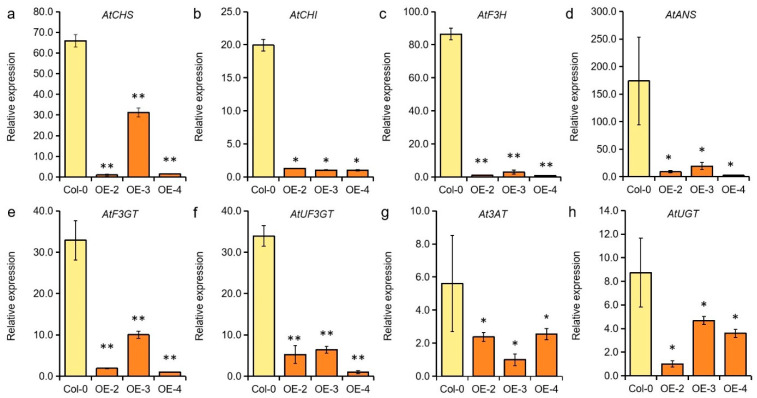
The expression levels of genes related to anthocyanin synthesis were detected in Col-0 and *GmMYB62* overexpression lines. The genes involved in anthocyanin synthesis show as (**a**–**h**): *chalcone synthase* (*AtCHS*, *At5G13930*), *chalcone isomerase* (*AtCHI*, *At3G55120*), *flavanone 3-hydroxylase* (*AtF3H*, *At3G51240*), *anthocyanidin synthase* (*AtANS*, *At4G22880*), *flavonoid 3-O-glucosyl-transferase* (*AtF3GT*, *At5G17050*), *UDP-glucose flavonoid 3-O-glucosyltransferase* (*AtUF3GT*, *At5G54060*), *anthocyanin acyl transferases* (*At3AT*, *At1G03940*), and *UDP-glycosyltransferases* (*AtUGT*, *At4G27560*). The relative expression levels of all genes were detected by quantitative reverse transcription PCR (qRT-PCR) using *AtEF-1α* as a housekeeping gene. Data represents the mean ± standard deviation of three replicates. Significant differences (* *p* < 0.05; ** *p* < 0.01) were determined by Student *t*-tests.

The anthocyanins are synthesized by a series of enzymes, including the chalcone synthase (CHS), chalcone isomerase (CHI), flavanone 3-hydroxylase (F3H), flavonoid 3′-hydroxylase (F3′H), flavonoid 3′5′-hydroxylase (F3′5′H), dihydroflavonol 4-reductase (DFR), leucoanthocyanidin dioxygenase (LDOX), anthocyanidin synthase (ANS), and UDP-glucose flavonoid 3-O-glucosyltransferase (UF3GT) [41]. To further reveal the genetic regulatory network of *GmMYB62* in the anthocyanin biosynthesis pathway, the expression levels of eight genes involved in anthocyanin biosynthesis were detected in Col-0 and *GmMYB62* overexpression lines. From the qRT-PCR results, the expression levels of *AtCHS*, *AtCHI*, *AtF3H*, *AtANS*, *AtF3GT* (*flavonoid 3-O-glucosyl-transferase*), and *AtUF3GT* in *GmMYB62* overexpression lines were down-regulated to 30% or even lower of the Col-0 (Figure 5a–f). For the other two genes, the *At3AT* (*anthocyanin acyl transferases*) and *AtUGT* (*UDP-glycosyltransferases*), which were also significantly down-regulated in *GmMYB62* overexpression lines when compared to Col-0 (Figure 5g,h). These results suggest that the *GmMYB62* transcription factor regulates anthocyanin accumulation and seed-coat pigmentation by inhibiting the expression of target genes that are involved in the anthocyanin synthesis pathway.

### 2.6. GmMYB62 Affects the Expression of Genes Involved in Cell Cycle and Seed Size Regulation in Arabidopsis

**Figure 6:**
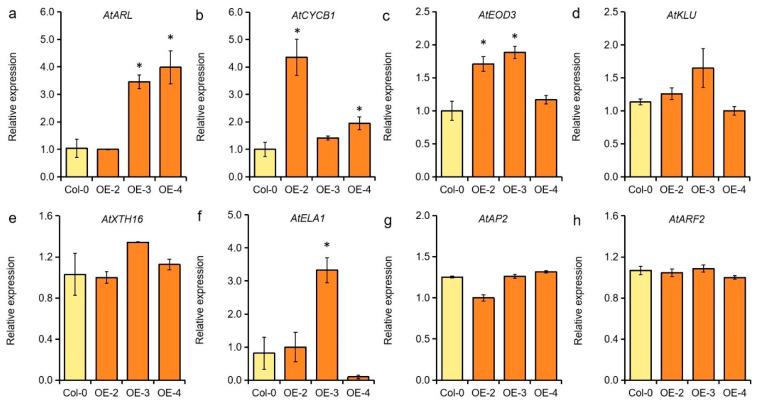
The expression levels of genes related to anthocyanin synthesis and seed size regulation were detected in Col-0 and *GmMYB62* overexpression lines. The genes involved in cell size and seed size regulation show as (**a**–**h**): *ARGOS-LIKE* (*AtARL*, *At2G44080*), *B-Type Cyclin* (*AtCYCB1*, *At2G26760*), *Enhancer of DA1-1* (*AtEOD3/CYP78A6*, *At2G46660*), *Cytochrome P450 KLUH* (*AtKLU/CYP78A5*, *At1G13710*), *Xyloglucan:xyloglucosyl transferase 16* (*AtXTH16*, *At3G23730*), *EUI-LIKE P450 A1* (*AtELA1/CYP714A1*, *At5G24910*), *APETALA2* (*AP2*, *At4G36920*), and *Auxin response factor* 2 (*ARF2 AT5G62000*). The relative expression levels of all genes were detected by quantitative reverse transcription PCR (qRT-PCR) using *AtEF-1α* as a housekeeping gene. Data represents the mean ± standard deviation of three replicates. Significant differences (* *p* < 0.05) were determined by Student *t*-tests.

To elucidate the regulatory mechanism of *GmMYB62* on seed size, the expression levels of eight genes that related to cell size and seed size regulation were also detected in Col-0 and *GmMYB62* overexpression lines. The Arabidopsis *ARGOS* genes transduce the auxin signal to affect cell proliferation of organs, thus controlling organ size [42], and the *ARGOS-LIKE* (*ARL*) contributed to change the cell size rather than cell number in Arabidopsis, indicating the *ARL* plays an important role in cell expansion-dependent organs [43]. The *AtARL* was up-regulated in *GmMYB62* overexpression lines compared to Col-0, and it could be positively affecting the width of seeds in Arabidopsis (Figure 4d and Figure 6a). The *B-type Cyclin* (*CYCB1*) was involved in the cell cycle to influence seed size in Arabidopsis [44], and its expression was increased in *GmMYB62* overexpression lines when compared to Col-0 (Figure 6b). The *enhancer of DA1-1* (*EOD3/CYP78A6*) controls seed size by promoting both cell proliferation and cell expansion in the Arabidopsis seed development [45]. Here, *AtEOD3* was also up-regulated in *GmMYB62* overexpression lines when compared to Col-0 (Figure 6c). Therefore, these three genes could be in the regulation pathway of seed size by balancing the seed length and width in transgenic Arabidopsis. In addition, the expression levels of the other five genes that are involved in seed size regulation were not significantly affected by the overexpression of *GmMYB62* in Arabidopsis (Figure 6d–h), indicating that *GmMYB62* may be involved in the other regulatory pathways of seed growth and development.

## 3. Discussion

### 3.1. GmMYB62 Is a R2R3-MYB Transcription Factor That Govern Anthocyanin Accumulation in Plants

Soybean seed-coat colors presented natural diversity that mainly included green, yellow, brown, and black. The seed-coat color was not only an evolutionary characteristic but also a sign of the commercial value of the soybean seeds. Compared with yellow seeds of soybean varieties, the black seeds contain higher anthocyanins and flavonoids within the seed coat [46,47]. In the anthocyanin synthesis and accumulation process, the R2R3-MYB transcription factors have been shown to function as activators and repressors, providing the capability for both reinforcement and feedback regulation [48,49].

In this study, the expression of *GmMYB62* continues to increase as the seed-coat color changes from yellow to brown to black in different soybean varieties (Figure 1a,b), indicating that *GmMYB62* could be involved in anthocyanin synthesis and accumulation in soybean. More than nine genetic quantitative trait loci (QTLs) have been identified that affect the seed-coat color formation in soybean, and the *R* locus was proposed to encode an R2R3-MYB protein (Glyma.09G235100) for the anthocyanin production in colored soybean seeds [20,50]. The *R* gene consists of multiple alleles, including *R* (black seed-coat), *r-m* (black spots on brown seed-coat), and *r* (brown seed-coat) [51]. Overexpression of four *MYB* genes (*GmMYB170/177203/211*) in Arabidopsis resulted in pale-brown seed-coat color and decreased anthocyanin content compared to Col-0, and their expression levels were up-regulated by *GmMYB5A* (*Glyma.14G154400*) in soybean [52]. Therefore, the different soybean MYBs contribute positive or negative regulatory effects to anthocyanin synthesis and accumulation.

The *GmMYB62* was highly expressed in soybean flower and pod tissues (Figure 2a), but it was different from the other three anthocyanin synthesis-related *MYB* genes of *GmMYBA1/2/5*. The *GmMYBA1* showed low expression in all tested tissues, including the stem, leaf, pod, and seed coat. The *GmMYBA2* showed high expression in the seed coat, while *GmMYBA5* presented high expression in the stem [36]. The GFP fluorescent signals in nuclei were observed for the *GmMYB62-GFP* construct in tobacco leaf epidermal cells (Figure 2b), indicating the nuclear localization of the *GmMYB62* protein, and the same subcellular distribution was also detected in GmMYBA1/2/5, GmMYB100, and GmMYB114 proteins that regulate anthocyanin and flavonoid synthesis, respectively [36,37,53]. The phylogenetic analysis classified these *MYB* homologous genes into two subclades, with *GmMYB62* falling into the dicot subclade (Figure 2c). Furthermore, the *GmMYB62* was also phylogenetically related to the subclade of MYB repressors (Figure S2), implying functional divergence in different MYBs [49], and offering insight into *GmMYB62* potential functions in regulating anthocyanin biosynthesis in soybean.

The *GmMYB62* was overexpressed in Arabidopsis, as validated by DNA level and transcription level (Figure 3b–d). The seeds in *GmMYB62* overexpression lines exhibited a pale-brown seed-coat color compared to the brown seed-coat color observed in Col-0 (Figure 4a). A similar feature in seed-coat color was also observed in four *MYB* genes (*GmMYB170/177/203/211*) overexpression lines in Arabidopsis [52]. Back to the *GmMYB62* in soybean, the *GmMYB62* could interact with other MYB activators/repressors or genes to regulate seed-coat color in soybean, and the different varieties and genetic backgrounds may also make the functional divergence of *GmMYB62* in soybean and Arabidopsis (Figure 1a,b and Figure 4a). The *MYB113* (*Vigun05g039500 and Phvul.008G038400*) was also mapped and identified to control seed-coat color in cowpea (*Vigna unguiculata*) and common bean (*Phaseolus vulgaris* L.) [54,55].

### 3.2. The Transcription Factor of GmMYB62 Specifically Regulates the Expression of Genes in the Pathway of Anthocyanin Synthesis

Different seed-coat colors were observed between *GmMYB62* overexpression lines and Col-0. To reveal the mechanism underlying the seed-coat color affected by *GmMYB62.* The anthocyanin content was detected, and the *GmMYB62* overexpression lines presented significantly lower anthocyanin content than Col-0 (Figure 4b). In transgenic Arabidopsis, the flavonol content was also significantly reduced in the *GmMYB100* (*Glyma.07G216000*) overexpression lines when compared with Col-0 [53]. However, the positive effect on anthocyanin accumulation was also contributed by other soybean *MYBs*, and overexpression of *GmMYBA1/2/5* induced significant enrichment of anthocyanins in tobacco leaves [36]. In addition, overexpression of *GmMYB114* also promoted anthocyanin synthesis and accumulation in soybean hairy roots [37].

The anthocyanins are synthesized by a series of enzymes located on the cytoplasmic face of the endoplasmic reticulum, like CHS, CHI, F3H, ANS, and so on in Arabidopsis. Therefore, the expression of eight genes involved in anthocyanins synthesis was detected, and all of them were significantly down-regulated by *GmMYB62* in transgenic Arabidopsis, including *AtCHS*, *AtCHI*, *AtF3H*, *AtANS*, *AtUGT*, *AtUF3GT* and so on (Figure 5a–h). For another negative regulator in soybean flavonoid biosynthesis, overexpression of *GmMYB100* also suppressed the expression of *CHS*, *CHI*, *F3H*, and *ANS* genes in soybean hairy roots [53]. On the contrary, the expression levels of *CHS*, *CHI*, *F3H*, *ANS*, and *UGT* genes were significantly up-regulated by overexpressing *GmMYB114* in soybean hairy roots to facilitate the synthesis of anthocyanins [37]. Overexpression of *GmMYBA1/2/5* in soybean hairy roots also greatly improved the expression levels of *F3H*, *F3′H*, *ANS*, *UGT*, and *DFR* genes [36]. Therefore, the soybean MYBs contribute positive or negative effects to anthocyanin synthesis by up-regulating or down-regulating the expression of genes involved in anthocyanin synthesis, respectively (Figure S2). When the expressions of these genes involved in anthocyanin synthesis were analyzed in the soybean accessions with different seed-coat colors, it was found that the *CHS*, *CHI*, *F3H*, *F3′H*, *ANS*, *UF3GT*, and *DFR* genes showed significantly high expression in Cheongja (black seeds) and Seum (black seeds) than in Kwangankong (yellow seeds) soybean accessions [56]. The seed-coat pigmentation was affected by anthocyanin synthesis and accumulation and finally regulated by *CHS*, *CHI*, *F3H*, *F3′H*, *ANS*, *UF3GT*, *DFR*, and *MYBs* genes.

### 3.3. R2R3-MYB Transcription Factor of GmMYB62 Was Involved in Seed Size Regulation

Seed size is another essential agronomic character in crop breeding, and identifying novel genes that govern soybean seed size could help us understand the genetic regulatory network of seed growth and development, and breed elite soybean cultivars. Several R2R3-MYB transcription factor family members have been discovered to regulate seed size, dormancy, and germination in plants. Overexpression of *AtMYB56* produced larger seeds than Col-0 by expanding endothelial cells and increasing cell number in the outer integument layer of the seed coat during the seed development [57]. The *OsMYBAS1*-overexpressing plants presented higher germination rates and root lengths than wild-type plants by significantly enhancing superoxide dismutase (SOD) enzyme activity and suppressing the accumulation of malondialdehyde (MDA) content in rice plants [58]. The *AtMYB96* contributes to the seed dormancy regulation by controlling the expression of *9-Cis-Epoxycarotenoid Dioxygenase* (*NCED2/5/6*) and *GA3ox1/GA20ox1* genes, which are involved in gibberellic acid (GA) and abscisic acid (ABA) metabolism [59].

Here, we found a soybean R2R3-MYB transcription factor of *GmMYB62* showed relatively higher expression in soybean varieties with small seed sizes (Figure 1c,d). Overexpression of *GmMYB62* in Arabidopsis produced smaller seeds with reduced length and increased width than Col-0 (Figure 4c,d), and the thousand-seed weight in *GmMYB62* overexpression lines were also significantly lower than Col-0 lines (Figure 4e). The reverse effect on seed size was governed by *AtMYB56*, and overexpression of *AtMYB56* resulted in bigger seeds, while the smaller seeds were observed in *atmyb56* mutant plants [57]. In *GmMYB62* overexpression lines, three genes of *AtARL*, *AtCYCB1*, and *AtEOD3* that are involved in cell expansion and seed size regulation were highly expressed than in Col-0 (Figure 6a–c). There was some discrepancy in the expression of *AtARL*, *AtCYCB1*, *AtEOD3*, and other tested genes observed in different transgenic lines, and this phenomenon could be caused by the genome insertion position of *GmMYB62* in different transgenic lines, or the expression level of *GmMYB62* in different transgenic lines, or the unknown environment facts that affect the genes expression in different transgenic lines.

The expression of *AtARL* was higher in *the atmyb56* mutant (smaller seed) than in Col-0 [57], suggesting that *AtARL* plays a crucial role in the smaller seed trait in *GmMYB62* overexpression lines. In soybean, the expression level of the *ARL* (*Glyma07G40380*) gene gradually increased with the seed development process, indicating its special role in soybean seed development (Figure S4a). Overexpression of *ZmCYCB1-1* in maize caused shorter kernel length and larger kernel width in some transgenic lines [60], and this was similar to the Arabidopsis seeds overexpressing *GmMYB62* in the present study (Figure 4a). In rice, the knockdown of *CYCB1-1* also resulted in an enlarged embryo with enlarged cells [61]. In soybean, the expression level of the *CYCB1* (*Glyma.14G037100*) gene gradually decreased with the seed development process (Figure S4b). The *EOD3* and *KLU* both are *CYP78A* subfamily members, and the expression of soybean *EOD3* and *KLU* also vary with the seed development process (Figure S4c,d). The soybean *GmCYP78A10* is also involved in seed size, and its different alleles significantly affect soybean seed width and thickness [62]. Overexpressing *GmCYP78A72* resulted in enlarged seeds both in transgenic soybean and Arabidopsis plants [63]. The *GmCYP78A57* was mainly expressed in soybean floral tissue and seed, and the expression levels of *GmCYP78A57* in different soybean cultivars showed positive correlations with 100-seed weight [64].

The *AtXTH16*, *AtELA1/CYP714A1*, *AtAP2*, and *AtARF2* that involved in cell expansion/proliferation and seed size regulation [57,63,65,66], showed inconsistent expression levels in *GmMYB62* overexpression lines or presented no significant difference between *GmMYB62* overexpression lines and Col-0 (Figure 6d–f). Like the *AtELA1* and *AtAP2*, play as negative regulators of seed size in Arabidopsis, were up-regulated in some *GmMYB62* overexpression lines (Figure 6f,g), and it could also be the reasons to generate smaller seed in *GmMYB62* overexpression lines. Overall, this study revealed that the *GmMYB62* was involved in seed size regulation in plants.

## 4. Materials and Methods

### 4.1. Plant Materials and Growth Conditions

The soybean plants were grown at the cycle of 16 h (26 °C)/8 h (22 °C) (day/night) in the greenhouse, and the different tissues were harvested at appropriate time. The soybean varieties L067 and L024 (yellow seed-coat), L144 and L120 (brown seed-coat), and L016 and L063 (black seed-coat) were planted in the experimental station, and the seeds were harvested for further analysis. The *Arabidopsis thaliana* was grown in the growth chamber at 22 °C under a 16 h light/8 h dark cycle. The *Nicotiana benthamiana* was grown in a growth chamber at 24 °C under a 16 h light/8 h dark cycle.

### 4.2. Bioinformatics Analysis of GmMYB62

The open reading frame and deduced amino acid sequence of *GmMYB62* (*Glyma.20G117000*) were searched by an online tool in the Phytozome database (https://phytozome-next.jgi.doe.gov/, accessed on 13 February 2024). The CDS of *GmMYB62* was amplified from Williams 82 using gene-specific primers according to the reference sequence in the Phytozome database (Table S1). The conserved domain was identified by SMART (https://smart.embl.de/, accessed on 13 February 2024).

### 4.3. Phylogenetic and Domain Analysis of MYB62 in Multispecies

The MYB domain (PF00249) from the GmMYB62 protein was used as a query in the Phytozome database and the MYB proteins from multiple species were selected. The MYB proteins from 19 species were used for multiple sequence alignments by ClustalW2. The unrooted phylogenetic tree was constructed by MEGA version 11 based on the Neighbor-Joining (NJ) method [67]. The MYB domain prediction for all 31 MYB proteins was performed by SMART. The phylogenetic tree of the MYB proteins as the activators or repressors in anthocyanin biosynthesis was constructed according to the previous study [49].

### 4.4. DNA, RNA Extraction and Quantitative Reverse Transcription PCR (qRT-PCR)

The genomic DNA was isolated from soybean and Arabidopsis tissues using the CTAB method [68]. Total RNAs were extracted from soybean and Arabidopsis tissues using FastPure Plant Total RNA Isolation Kit (Vazyme, Nanjing, China), and the cDNA was synthesized by HiScript III RT SuperMix for qPCR (+gDNA wiper) (Vazyme, Nanjing, China). The primers for qRT-PCR were designed using Primer Premier 5 software and Primer-BLAST (https://www.ncbi.nlm.nih.gov/tools/primer-blast/, accessed on 13 February 2024) (Table S1). To detect the transcript level of *GmMYB62* in seed-coat and seed, the soybean seed coat was obtained when the soybean seeds matured, and the rest seed without a coat was also harvested. Then, the RNA was extracted from the seed coat and seed to identify the expression level of *GmMYB62*. The *GmEF-1α* (*Glyma.17G186600*) and *AtEF-1α* (*At01G07940*) were used as reference genes for semi-quantitation reverse transcription PCR (RT-PCR) and qRT-PCR in soybean and Arabidopsis, respectively [69,70]. The ChamQ Universal SYBR qPCR Master Mix (Vazyme, Nanjing, China) was used in qRT-PCR through the Roche LightCycler 96 Instrument (Roche Diagnostics, Basel, Switzerland). Each experiment was performed in three biological repetitions and the expression levels of all candidate genes were analyzed by the 2^−CT^ methods [24,71].

### 4.5. Subcellular Localization

The CDS of *GmMYB62* was amplified and in-fusion expressed with a green fluorescent protein (GFP) under the cauliflower mosaic virus (CaMV) 35S promoter (*35S::GmMYB62-GFP*) in the backbone vector of pBinGFP4, and the empty vector containing *35S::GFP* were used as controls. The Agrobacterium-mediated transient transformation was performed on leaves from 4-week-old tobacco (*Nicotiana benthamiana*) plants using a method described previously [72]. The subcellular localization of the GmMYB62 protein was observed with a laser-scanning confocal microscope (Zeiss LSM900, Jena, Germany).

### 4.6. Generation and Selection of GmMYB62-Overexpressing Transgenic Arabidopsis Plants

The CDS of *GmMYB62* is cloned into the pBinGFP4 vector to generate *35S::GmMYB62* plasmid using restriction enzyme cutting site of *BamH* I by one step cloning kit (VAZYME, Nanjing, China). The *35S::GmMYB62* plasmid was introduced into Arabidopsis using Agrobacterium-mediated transformation following the floral dip method [73]. Transgenic lines were selected on 1/2 MS medium supplemented with kanamycin (25 mg/L) and then identified by PCR. The seedlings with resistance to antibiotics were selected and transferred to the plant in sterile sand in the growth chamber. The leaves of the selected seedlings were collected for RNA extraction, and the RT-PCR and qRT-PCR were used for the expression detection of *GmMYB62* in transgenic Arabidopsis plants. The T_3_ generation of homozygous transgenic lines with a high expression level of the *GmMYB62* was used for the experiment.

### 4.7. Seed Size and Weight Measurement

The mature seeds of soybean and Arabidopsis were harvested and dried for one week at an appropriate temperature. The seeds from *GmMYB62* overexpression lines and Col-0 were photographed by a Zeiss microscope (Axio Zoom.V16, Baden, Germany), and the ImageJ software was used to measure the length and width of the seeds. The soybean and Arabidopsis seed width was measured from the hilum facade to the opposite side. The seed mass of soybean (hundred-seed) and Arabidopsis (thousand-seed) were weighed by an electronic analytical balance (FA3004, China).

### 4.8. Relative Anthocyanin Content Measurement

Total anthocyanin content was extracted and quantified in Arabidopsis plants as follows. The plant tissues (100 mg) were ground in liquid N_2_ and incubated in methanol (1.00 % HCl) at 4 °C for 24 h. After centrifugation (10 min, 10,000 rpm, 4 °C), the absorbance of the supernatants was measured at 530 and 657 nm, and the relative anthocyanin content was calculated with the formula [A_530_ − (0.25 × A_657_)] [74]. One anthocyanin unit was defined as the absorbance in 1 mL extraction solution.

### 4.9. Statistical Analysis

Statistical comparisons were conducted through Student’s *t*-test by SPSS 22.0 (SPSS Inc., Chicago, IL, USA). The significant differences were denoted as * *p* ≤ 0.05, ** *p* ≤ 0.01.

## 5. Conclusions

In this study, the correlations between the expression of *GmMYB62* and seed-coat pigmentation and seed size were observed in soybeans, indicating the crucial role of *GmMYB62* in the formation of soybean agronomic traits. The GmMYB62 and other R2R3-MYB proteins were relatively conserved between the monocot and dicot, and the GmMYB62 also clustered with MYBs that act as repressors in the anthocyanin biosynthesis and accumulation. Overexpression of *GmMYB62* in Arabidopsis produced seeds with pale-brown coats, while the dark-brown seed coat was observed in Col-0. Moreover, the seeds from *GmMYB62* overexpression lines showed shorter length, larger width, and lower thousand-seed weight than that in Col-0. The expressions of eight genes that are involved in anthocyanin synthesis, like *CHS*, *CHI*, *F3H*, and *ANS*, were down-regulated in *GmMYB62* overexpression lines compared to Col-0. Three genes, *ARL*, *CYCB1*, and *EOD3*, which control cell expansion and proliferation, were also up-regulated in *GmMYB62* overexpression lines. In summary, the *GmMYB62* was involved in seed-coat color and seed size regulation by affecting the expression levels of genes related to anthocyanin synthesis and cell expansion and proliferation, respectively. The overexpression and CRISPR/Cas9 mutant of GmMYB62 in soybeans should be obtained in the future to further confirm its role in soybean seed-coat pigmentation and seed size regulation.

## Data Availability

All data to support the conclusions of this study are included in the main text and the Supplementary Data.

## Supplementary Materials

The following supporting information can be downloaded at: https://www.mdpi.com/article/10.3390/ijms26083457/s1.
