# Supplementary material for: A R2R3-MYB Transcription Factor of GmMYB62 Regulates Seed-Coat Color and Seed Size in Arabidopsis
Source: Int J Mol Sci. 2025 Apr 8;26(8):3457. doi: 10.3390/ijms26083457 (PMC12026657; doi:10.3390/ijms26083457)
Supplement: Supplementary file 1 [file ijms-26-03457-s001.zip › Figure S1-4.docx]

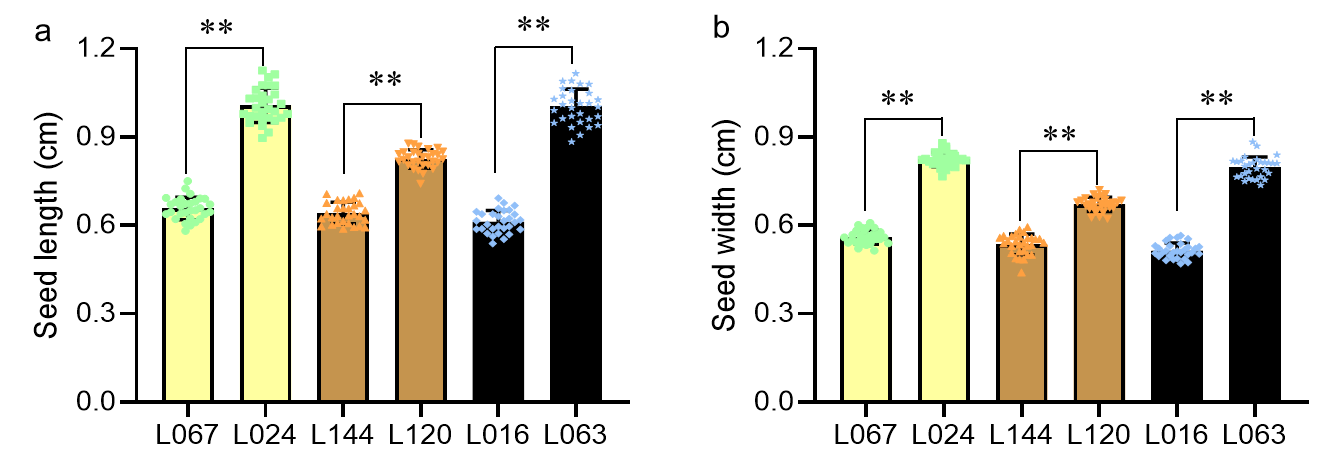


**Figure S1** Phenotypes of soybean seed length and width in six soybean varieties with different seed-coat color. **a** The seed length in six soybean varieties. **b** The seed width in six soybean varieties. Data represents the mean ± standard deviation of 30 replicates. Significant differences (**P* < 0.05; ***P* < 0.01) were determined by Student *t*-tests.


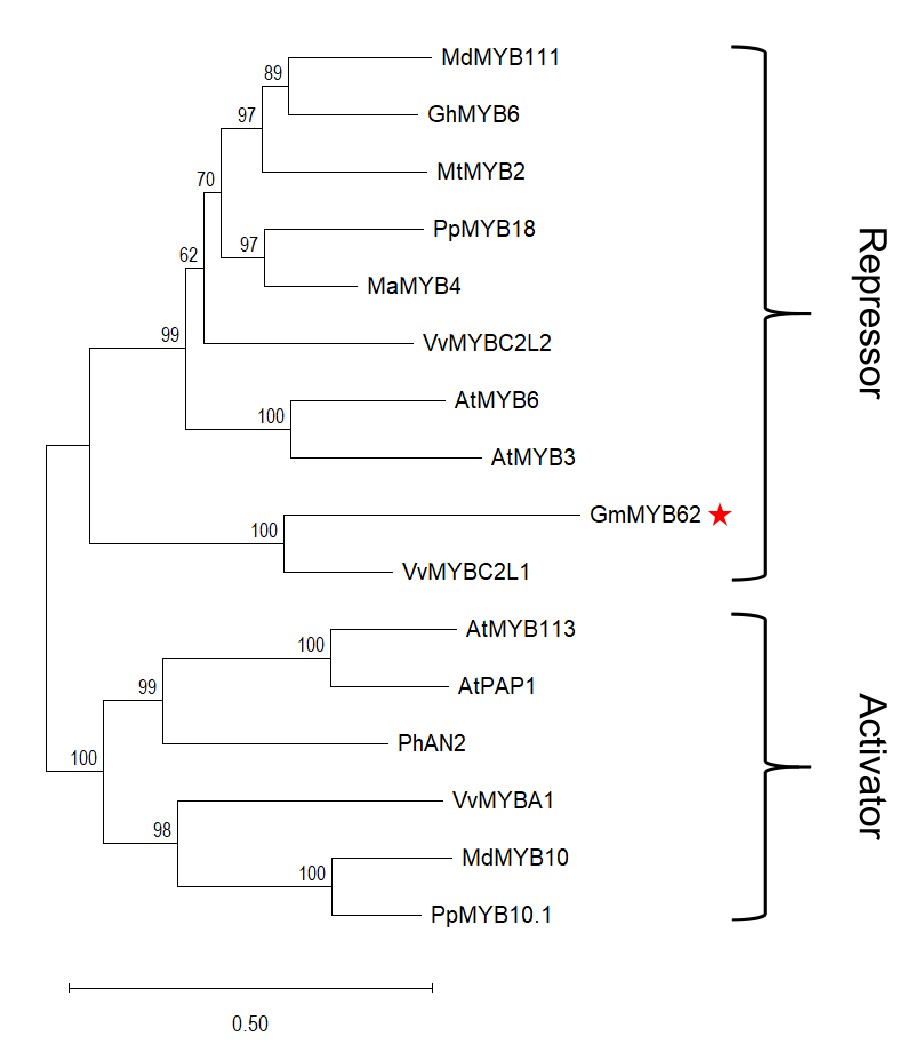


**Figure S2** Phylogenetic analysis of R2R3-MYB proteins from soybean and other species as the activator or repressor in anthocyanin biosynthesis. These R2R3-MYB proteins from other species work as the activator or repressor in anthocyanin biosynthesis were classified according to Zhou *et al* study (Please check Zhou et al., 2019 in reference of this article). The phylogenetic tree was constructed using the Neighbor-Joining method with 1,000 bootstrap replicates by MEGA version 11.


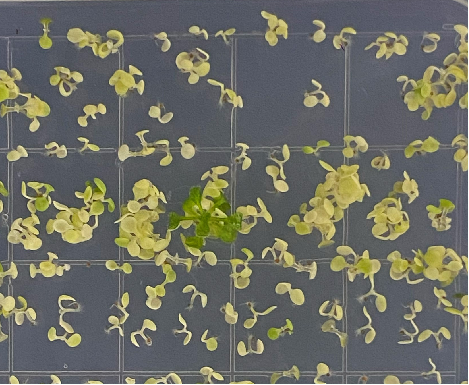


**Figure S3** Screening of *GmMYB62* transgenic Arabidopsis lines by Kanamycin in 1/2 MS medium. The work solution of Kanamycin is 25 mg/L.


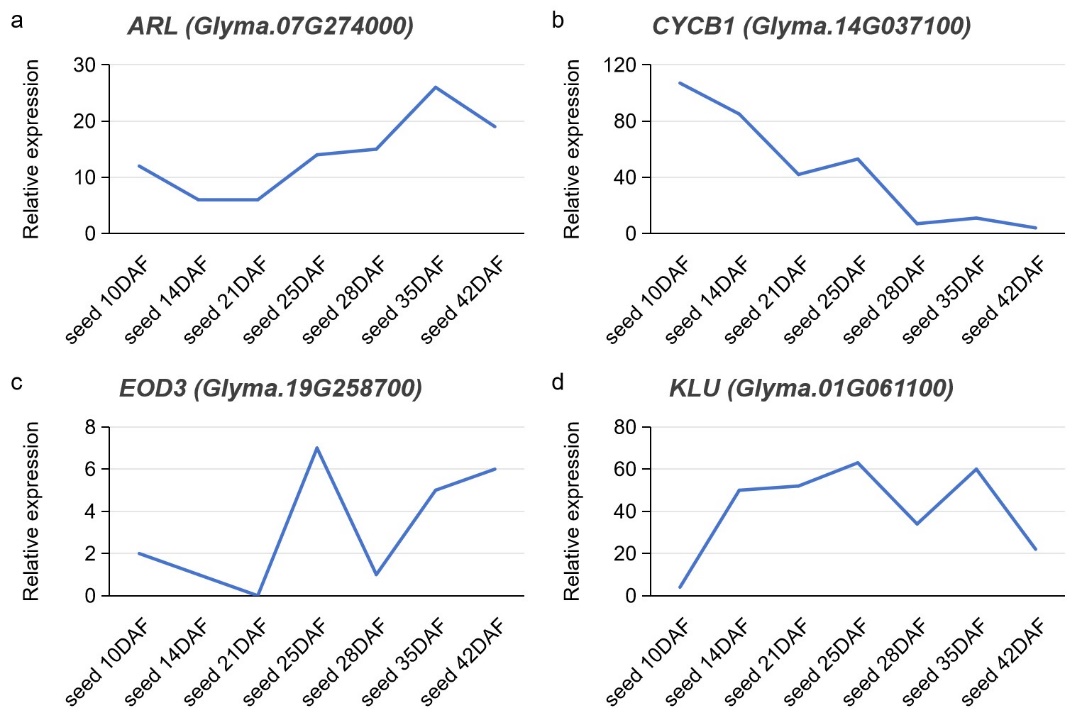


**Figure S4** The relative expression level of genes related to cell expansion and proliferation in soybean seed during different stages. The gene expression data was obtained from Soybase (https://www.soybase.org/). *ARL: ARGOS-LIKE*; *CYCB1: B-Type Cyclin*; *EOD3/CYP78A6: Enhancer of DA1-1*; *KLU/CYP78A5: Cytochrome P450 KLUH*; DAF: Day after flower.
